# Genotypic and phylogeographic insights into a pre-epidemic variant of Wesselsbron virus detected in sylvatic *Aedes mcintoshi* from Semuliki Forest, Uganda

**DOI:** 10.1128/spectrum.00914-24

**Published:** 2024-11-12

**Authors:** Georg Joachim Eibner, Selina Laura Graff, Christian Hieke, James Robert Ochieng, Anne Kopp, Christian Drosten, Julius Lutwama, Innocent Bidason Rwego, Sandra Junglen

**Affiliations:** 1 Institute of Virology, Charité–Universitätsmedizin Berlin, corporate member of Freie Universität Berlin and Humboldt-Universität zu Berlin, Berlin, Germany; 2 Department of Zoology, Entomology and Fisheries Sciences, Makerere University, Kampala, Uganda; 3 Department of Arbovirology, Uganda Virus Research Institute (UVRI), Entebbe, Uganda; 4 Department of Biosecurity, Ecosystems and Veterinary Public Health, Makerere University, Kampala, Uganda; Universidade Federal do Rio de Janeiro, Rio de Janeiro, Brazil

**Keywords:** Wesselsbron virus, flavivirus, mosquito, arbovirus, phylogeographic spread

## Abstract

Wesselsbron virus (WSLV) is a neglected mosquito-borne virus within the yellow fever subgroup in the genus *Orthoflavivirus* of the *Flaviviridae* family. Despite being primarily a veterinary pathogen able to cause stillbirths, congenital malformations, and mortality in ruminants, WSLV also infects humans, causing a usually self-limiting febrile illness, or may lead to neurological complications in rare cases. WSLV causes sporadic outbreaks in Southern Africa, but findings in mosquitoes from other African countries suggest a wider distribution. Here, we report the detection and isolation of WSLV from an *Aedes mcintoshi* mosquito collected in a pristine ecosystem within Semuliki National Park, western Uganda. The detected strain M5937-UG-2018 was impaired in infectivity, replication, and production of infectious particles in cell lines derived from different hosts compared to an epidemic reference strain, SA H177. Full-genome sequencing by next-generation sequencing from the mosquito homogenate revealed a maximum nucleotide identity of 98.1% to a WSLV isolate from a human sample collected in South Africa in 1996. M5937-UG-2018 grouped in phylogenetic analyses with strains from South Africa and Senegal. Reconstruction of the temporal and spatial dispersal of WSLV across Africa estimated a likely origin of WSLV in South Africa in the early 19th century and spread in Southern Africa in the following decades. Long-distance movement toward Western and Eastern Africa was modeled to have occurred in the early 21st century. However, displacing the origin of M5937-UG-2018 did not decrease the likelihood of the model supporting the hypothesis that WSLV is widely distributed in Africa.

**IMPORTANCE:**

WSLV is a neglected mosquito-borne virus causing teratogenicity in ruminants and febrile illness in humans. WSLV is mainly endemic to Southern Africa, but findings in other regions suggest a wider distribution on the continent. Knowledge of the distribution of WSLV is impaired as differential diagnostics are rarely performed in livestock and humans presenting with symptoms compatible with WSLV infection. Our work investigating viral infections in mosquitoes from a remote tropical rainforest region demonstrates that WSLV is endemic in Uganda. The isolated virus was less infective and showed lower replication ability *in vitro* compared to an epidemic isolate from South Africa. Phylogeographic reconstruction of spatial and temporal movements, along with the displacement of the origin of the newly detected strain**,** suggests that WSLV may be widely distributed across Africa. Our data show that the geographic distribution of WSLV and its impact on human and animal health are likely underestimated.

## INTRODUCTION

Mosquito-borne flaviviruses (family *Flaviviridae*, order A*marillovirales*) such as Zika virus or West Nile virus have demonstrated great potential for global epidemic spread over the past decades (1, 2). While these viruses are well studied due to their clinical relevance, other related viruses have received comparably less attention despite their capacity to inflict momentous outbreaks. Wesselsbron virus (WSLV) is a member of the yellow fever virus subgroup within the mosquito-borne flaviviruses. Multiple outbreaks in South Africa and neighboring countries were documented in the 1950s and subsequent decades (3–5). WSLV infects several livestock species, such as sheep, cattle, and goats, as well as humans (6–9). Common symptoms of disease include abortions in pregnant sheep and goats (10, 11), cerebellar hypoplasia in calves (12), and neurological dysfunction in horses (13). Particularly, newborn lambs and goats suffer from high mortality rates of up to 27% (14). Typical symptoms in humans include fever, headache, chills, body aches, insomnia, rash, and, in rare occasions, mild visual and cognitive abnormalities (3). Liver involvement has also been observed but is rather unspecific, similar to the previously mentioned symptoms (15). Consequently, misdiagnosis and insufficient awareness of WSLV circulation may impair the identification of the causative pathogen of local outbreaks. While only 29 acute human cases have been described between 1955 and 1996, seroprevalence data from several African countries suggest higher infection rates (8, 16–20). The most recent outbreaks were reported from South Africa in 2010–2011 and Senegal in 2013, with two laboratory-confirmed human cases, respectively (8, 9).

A large variety of mosquito species, mainly of the genus *Aedes*, seems to be involved in WSLV transmission (21, 22). Floodwater mosquitoes such as *Aedes circumluteolus* and *Aedes mcintoshi* have been identified as the main vectors for WSLV in South Africa and Zimbabwe (8) and *Aedes vexans* in Mauretania (23). WSLV has also been found in *Culex sp*. and *Anopheles coustani* mosquitoes in Kenya (24), as well as in *Aedes gibbinsi* and *Aedes tricholabis* in Uganda (25). To date, there is little knowledge on the endemic maintenance cycle of WSLV. Numerous vertebrate hosts, such as camel, wild ungulates, rodents, ostriches, and other wild birds, were found to be infected with WSLV, but it is not clear which wildlife species serve as amplification hosts (3, 8, 9, 26). Apart from Southern Africa, no disease outbreaks were reported from other regions in Africa, although isolated findings of WSLV in mosquitoes from West Africa [Cameroon, Nigeria, Ivory Coast, Senegal, and Mauritania (8, 22, 23)] and East Africa [two reported detections in field-caught mosquitoes from rural areas in Kenya (24) and Uganda (25)] indicate a wider distribution of the virus. Since little monitoring or differential diagnosis is conducted across Africa, knowledge on the geographic distribution and impact of WSLV on animal and human health is limited.

The genome of WSLV consists of single-stranded positive-sense RNA, is approximately 10,800 nucleotides in length, and shares the same genome organization as other flaviviruses (27). Its single open reading frame encodes for a polyprotein which is post-translationally processed into three structural and seven non-structural proteins (28).

Here, we aimed to gain a better understanding of the geographic distribution of WSLV in East and Central Africa. We collected mosquitoes from the primary rainforest of Semuliki National Park in western Uganda, which is contiguous with the primary lowland rainforest of the Congo basin in the Democratic Republic of Congo. Furthermore, we were interested to investigate whether WSLV strains from this region would exhibit genetic and phenotypic differences compared to strains from Southern Africa.

## RESULTS

### Detection of WSLV in *Aedes mcintoshi* from a rainforest in Uganda

In total, 27,405 mosquitoes were collected in the Semuliki National Park area in 2018 and 2019 and were tested in pools (*n* = 343) for infection with WSLV by quantitative PCR (qPCR). WSLV was detected in one mosquito pool. Subsequent testing of individual mosquitoes from the positive pool revealed that one female *Aedes mcintoshi* mosquito collected in August 2018 within the sylvatic environment of Semuliki National Park (0.82°N, 30.15°E) was positive for WSLV.

The entire WSLV genome was sequenced from the individual mosquito specimen using high-throughput sequencing on an Illumina MiSeq platform. Genome termini were confirmed by rapid amplification of cDNA ends by PCR. The genome of 10,808 nucleotides was assembled from 234,905 reads with a coverage ranging from 688 to 3,064. The coding sequence consists of 10,218 nucleotides with a 5′-non-coding region (NCR) of 118 nucleotides and a 3′-NCR of 472 nucleotides. The genome organization, including the polyprotein and its cleavage sites, is identical to other WSLV strains. The genome sequence of the newly detected WSLV strain M5937-UG-2018 was compared to the complete genomes of all other publicly available WSLV strains (*n* = 8). M5937-UG-2018’s pairwise nucleotide and amino acid identities with other WSLV strains varied from 93.2% to 98.1% and 98.9% to 99.7%, respectively (Table S1). The highest nucleotide and amino acid identities of 98.1% and 99.7%, respectively, were with the South African strain AV259, isolated from a human sample in 1996 [accession number JX423784 (5)].

We examined all available WSLV genomes to identify nucleotide substitutions. In total, we found 26 non-synonymous nucleotide substitutions between strain M5937-UG-2018, identified in this study, and the epidemic prototype strain from South Africa H177 (Table 1). Notably, strain H177 exhibits 22 unique non-synonymous nucleotide substitutions (Table 1).

**TABLE 1:** Amino acid substitutions between M5937-UG-2018 and the epidemic strain H177^*a*^

Protein	M5937	H177
C	L_113_*	I_113_
E	V_323_	I_323_
	S_351_*	T_351_
	S_384_	G_384_
	V_433_*	E_433_
	Q_516_*	E_516_
	V_757_*	I_757_
NS1	V_977_	I_977_*
	R_1024_*	K_1024_
	R_1047_*	K_1047_
NS2A	L_1243_*	I_1243_
	M_1286_*	V_1286_
	V_1287_*	A_1287_
	I_1305_*	V_1305_
NS2B	I_1458_*	T_1458_
NS3	I_1819_*	V_1819_
	K_1839_*	R_1839_
NS4B	N_2460_*	D_2460_
NS5	V_2751_*	I_2751_
	P_2771_*	T_2771_
	R_2790_*	K_2790_
	O_3051_*	E_3051_
	V_3155_*	A_3155_
	V_3333_*	I_3333_
	V_3346_	I_3346_
	E_3396_*	D_3396_

^*a*^ Unique amino acid substitutions compared to the eight publicly available WSLV genomes are marked with gray shadings. Positions where all other genomes differ from one of the strains but share the same amino acid are marked with an asterisk (*). Protein abbreviations: C, capsid; E, envelope; NS1, non-structural protein 1; NS2A, non-structural protein 2A; NS2B, non-structural protein 2B; NS3, non-structural protein 3; NS4B, non-structural protein 4B; NS5, non-structural protein 5.

### Phylogenetic relationship and phylogeographic spread of M5937-UG-2018

**Fig 1:**
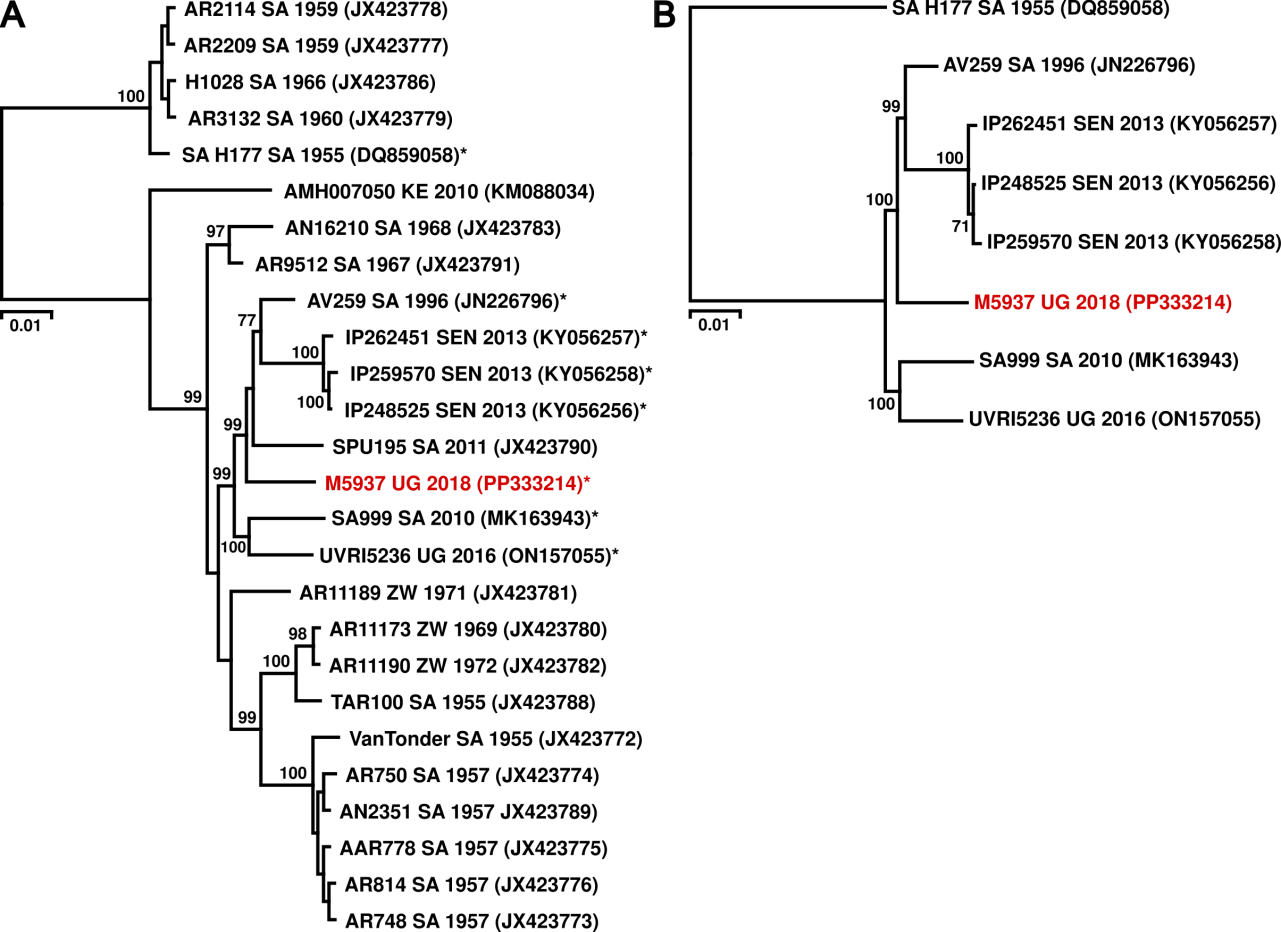
Phylogenetic relationship of WSLV M5937-UG-2018. (**A**) A Bayesian phylogenetic tree was generated using 18 partial NS5 sequences (length = 952 nt) and 8 complete WSLV genome sequences, the latter indicated by an asterisk. The newly discovered strain is highlighted in red. (**B**) Maximum likelihood phylogenetic tree based on a translational alignment of eight complete WSLV coding sequences.

Phylogenetic analyses based on all available WSLV sequences (*n* = 26), including partial sequences, placed M5937-UG-2018 in a diversified clade with strains from South Africa and Senegal using MrBayes (21) for Bayesian phylogenetic inference (Fig. 1A). This tree topology was confirmed using a maximum likelihood approach via IQ-TREE 2 (29), based on complete coding sequences only (*n* = 8, Fig. 1B).

We next performed selection analyses to identify specific regions of the WSLV genomes which were subject to selection pressure during their evolution. Testing for positive selection based on the alignment of complete WSLV genomes using mixed effects model of evolution (MEME) (30) revealed seven codon sites that were subject to episodic diversifying selection (*P* < 0.05) (Table 2). The WSLV phylogenetic tree is divided in a clade containing WSLV sequences from South Africa isolated in the 1950s that were responsible for a large outbreak (including the epidemic strain H177) and a second clade containing all other sequences. We tested if positive selection occurred on the former clade represented by strain H177. However, the adaptive branch-site random effects likelihood model (aBSREL) (31) did not find significant evidence for episodic diversifying selection (*P* = 0.075).

**TABLE 2:** Test results for episodic diversifying selection via MEME^*a*^

Nt pos	LRT	MEME LogL	FEL LogL	Variation *P*	*P* value
6897	15.05	−19.98	−12.00	<0.01	<0.01
6894	8.06	−19.23	−15.19	0.02	0.01
9153	8.79	−14.76	−10.92	0.02	0.01
4011	5.04	−12.81	−10.35	0.09	0.04
4611	5.00	−12.70	−10.58	0.12	0.04
4845	4.90	−13.02	−11.02	0.14	0.04
8232	4.76	−12.96	−11.04	0.15	0.04

^*a*^ FEL LogL, site log likelihood under the FEL model; LRT, likelihood ratio test for episodic diversification; MEME LogL, site log likelihood under the MEME model; Nt pos, nucleotide positions of the respective codons within the CDS of WSLV found to underlie positive selection pressure (*P* < 0.05); variation *P*, asymptotic *P* value if evidence exists for dN/dS across branches of the phylogenetic tree.

**Fig 2:**
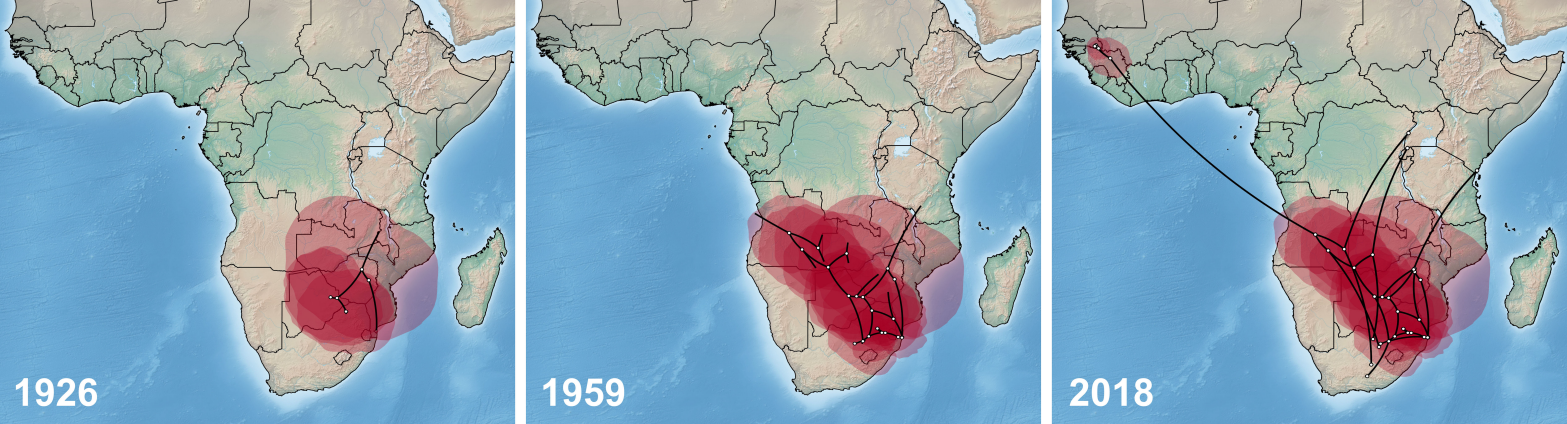
Bayesian reconstruction of WSLV spatiotemporal diffusion across Africa based on available partial and complete genome sequences of 26 WSLV strains and their location and date of origin. WSLV geographic spread is shown for the three time points indicated in white. Black lines show the spatial projection of the maximum clade credibility tree, with each node mapped to its known (external node) or estimated (internal node) location. Red clouds represent statistical uncertainty in the estimated locations of WSLV lineages (95% HPD regions).

Phylogeographic spread reconstructions placed the root of WSLV within the northeast of South Africa in the early 18th century (Fig. 2). In the early 19th century, the subsequent supraregional diffusion spans over a core area surrounding approximated internal tree nodes in northern South Africa, Botswana, Zimbabwe, and southern Mozambique. From there, WSLV was estimated to have first protruded further north with lower density followed by accelerated expansion northwestward, reaching the coast of Angola by the end of the 1950s. Thereafter, WSLV is predicted to have dispersed across large parts of southern Africa in the 1960s, sparing the central to southwestern areas of Namibia and South Africa. In the following decades, the WSLV density increased in these areas, with further spread throughout Zimbabwe and southern South Africa in the 1970s. Originating from two paraphyletic internal tree nodes placed in eastern Angola and central Zambia, long terminal branches sprout north, to the areas of detection of the southern Ugandan strain UVRI5236, as well as of the strain M5937-UG-2018 described here. By the early 2000s, WSLV is predicted to have reached Uganda, Kenya, and West Africa while continuously occurring in central South Africa.

To explain the detection of WSLV in a pristine rainforest area in Uganda, two hypotheses were formulated: (i) M5937-UG-2018 belongs to an enzootic/sylvatic lineage of WSLV that has existed in Central Africa for an extended period, or (ii) M5937-UG-2018 was introduced to Uganda from Southern Africa, as suggested by the phylogeographic analyses. To assess the likelihood of these hypotheses, we employed arbitrary coordinates to represent the place of origin for WSLV M5937-UG-2018 and evaluated the strength of evidence using Bayes factors. Here, the Bayes factors serve as the ratio between the likelihood of one hypothesis and the likelihood of another hypothesis. A Bayes factor close to 1 indicates that the alternative hypothesis (introduction from Southern Africa) does not significantly outweigh the baseline hypothesis (originating from Uganda/Central Africa). Our findings did not support the hypothesis that WSLV M5937-UG-2018 was introduced into the sampling area. All arbitrary placements of WSLV M5937-UG-2018 yielded similar results close to 1 (Bayes factors ranging from 0.9999 to 1.0002 and marginal likelihood estimates ranging from −21,330.62 to −21,325.44; see Table 3). However, when we placed the origin of WSLV M5937-UG-2018 in Berlin, Germany, the Bayes factor deviated more than in any of the other alternative hypotheses (Fig. S1). Overall, our analyses provide strong support for the baseline hypothesis: WSLV M5937-UG-2018 is likely a descendant of an ancestral WSLV lineage from Central Africa.

**TABLE 3:** MLEs of 12 Bayesian phylogeographic models^*a*^

Model no.	Lat.	Long.	Country	MLE	BF
1	0.82	30.15	Uganda	−21,329.727	1.0
2	−34.2	18.38	South Africa	−21,326.817	1.0001
3	−27.6	28.63	South Africa	−21,325.386	1.0002
4	−24.6	25.91	Botswana	−21,325.022	1.0002
5	−22.7	17.22	Namibia	−21,325.444	1.0002
6	−17.4	22.48	Angola	−21,327.461	1.0001
7	−11.7	27.51	DR Congo	−21,326.776	1.0001
8	−4.26	28.16	DR Congo	−21,330.091	0.9999
9	13.1	14.12	Chad	−21,330.006	0.9999
10	12.66	12.6	Nigeria	−21,330.244	0.9999
11	13.03	−12.03	Senegal	−21,330.616	0.9999
12	52.58	13.32	Germany	−21,341.349	0.9994

^*a*^ Model 1 is the baseline model using the original geographic coordinates of the novel strain. Subsequently, the coordinates were arbitrarily altered and compared to the baseline model by calculating the respective BF. BF, Bayes factor.

### M5937-UG-2018 displays impaired infectivity, replication, and release of infectious particles

**Fig 3:**
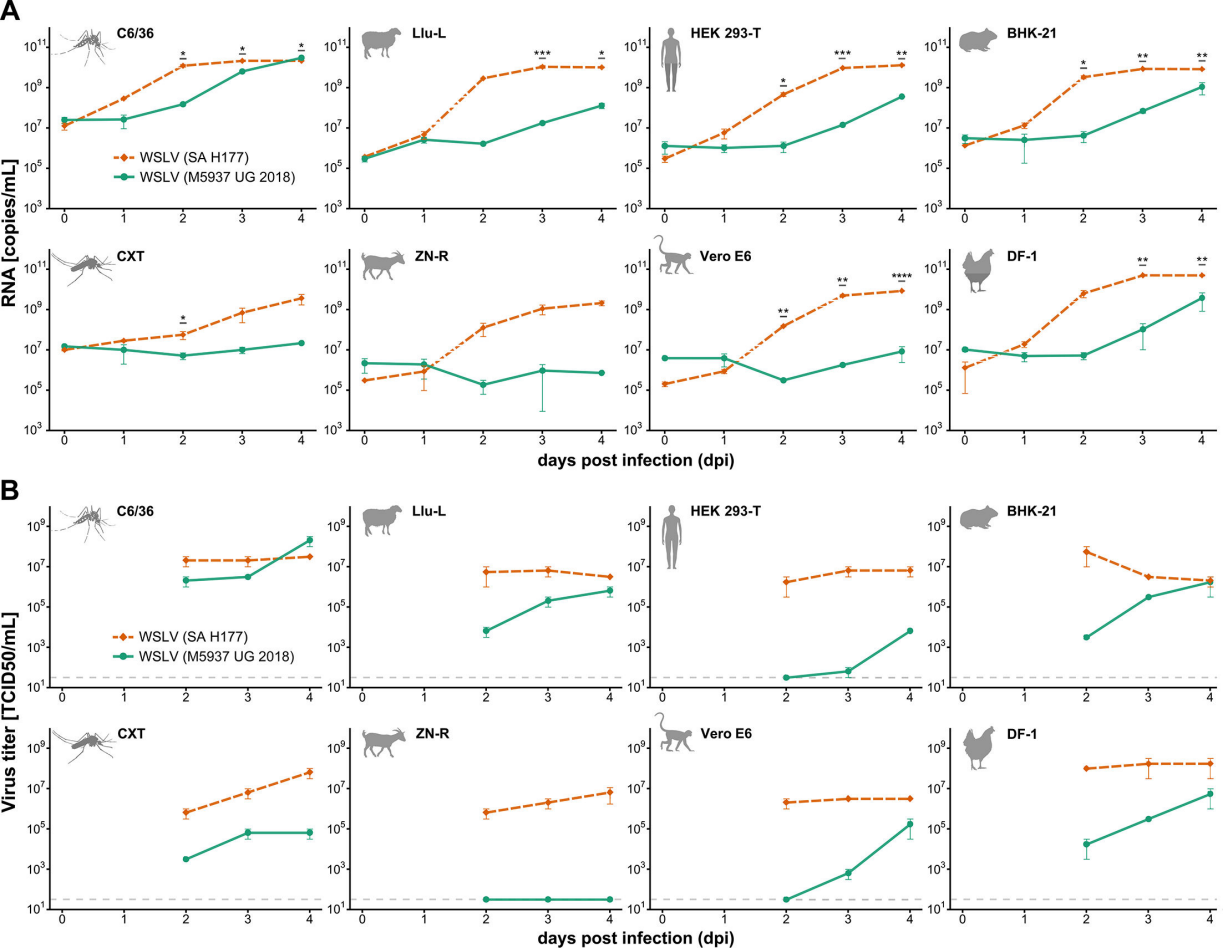
Viral growth of WSLV M5937-UG-2018 and the reference strain SA H177. Cell lines were infected in duplicate with M5937-UG-2018 or SA H177 at a multiplicity of infection of 0.1. Viral RNA copies per milliliter were quantified by real-time PCR at the indicated time points (**A**). Statistically significant differences between the mean RNA copy numbers of the two WSLV strains as determined by a two-sample Student’s *t*-test are denoted by asterisks. The number of infectious particles was determined by TCID_50_ titration in combination with quantification of number of genome copies in serial dilutions (**B**), with the gray dashed line representing the limit of detection.

**Fig 4:**
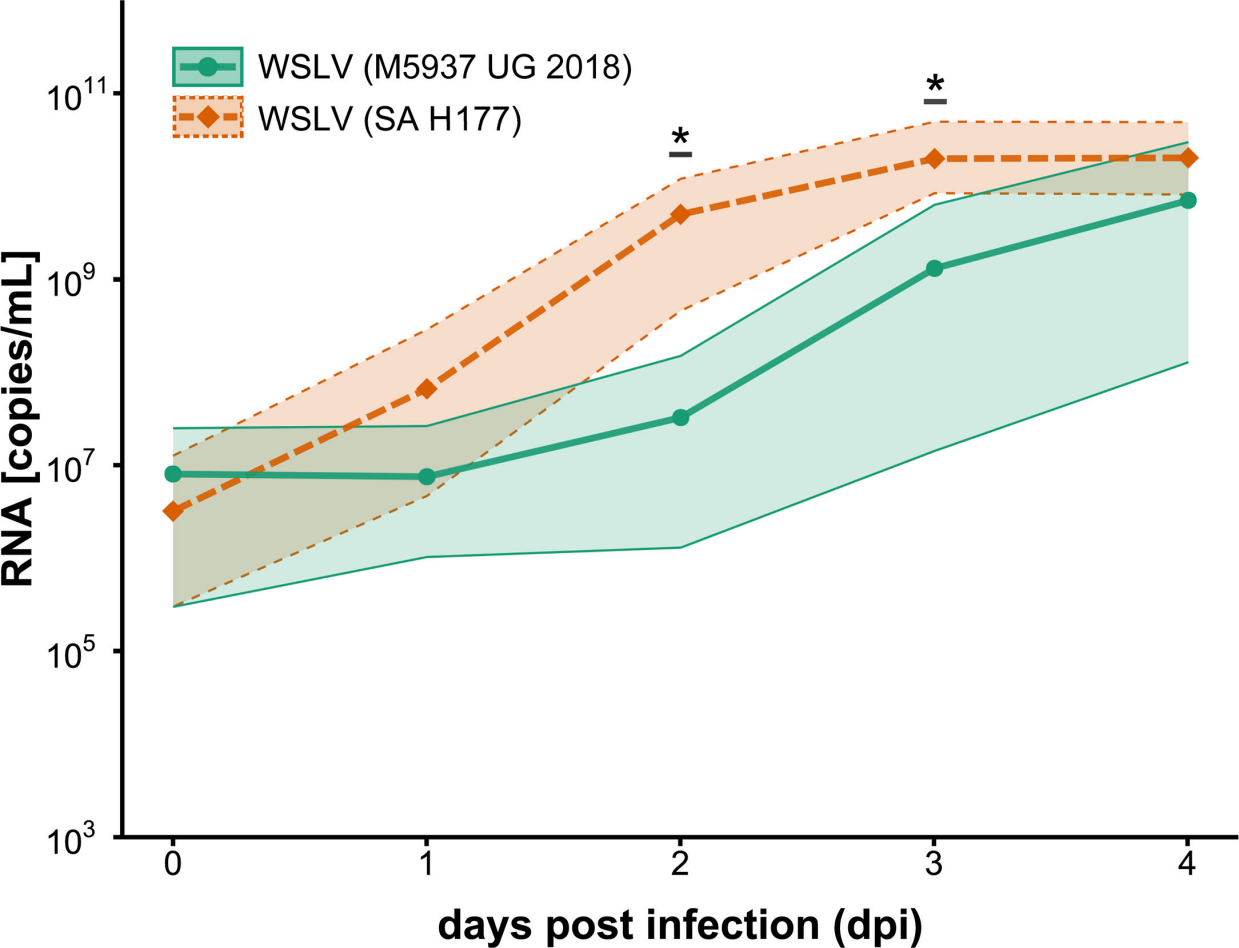
Mean viral growth of WSLV M5937-UG-2018 and the epidemic reference strain SA H177 in five different cell lines (C6/36, Llu-L, HEK 293-T, BHK-21, and DF-1). Mean values (bold curve) were retrieved from Fig. 3. Thin lines and shading indicate the 95% confidence interval. Asterisks (*) indicate statistically significant differences between the mean copy numbers of both strains, as determined by a two-sample Student’s *t*-test (*P* < 0.05).

As M5937-UG-2018 was found in a sylvatic environment and may represent an enzootic virus, we sought to test its phenotypic characteristics. Virus isolation attempts were successful in C6/36 cells but not in Vero E6 cells. M5937-UG-2018 induced weak cytopathic effects (CPES) in C6/36 cells, and viral replication was confirmed via qPCR. The genomes of M5937-UG-2018 and SA H177 from the generated viral stocks were sequenced and were identical to the sequences derived from the mosquito homogenate and from the sequence deposited at National Center for Biotechnology Information (NCBI) (NC_012735), respectively. Growth kinetics with M5937-UG-2018 and the prototype epidemic strain SA H177 were performed in two mosquito (C6/36 and CXT) and six vertebrate cell lines (BHK-21, DF-1, HEK 293 T, Llu-L, Vero E6, and ZN-R). M5937-UG-2018 induced CPE in C6/36 but not in Vero E6 cells, whereas SA H177 induced CPE in Vero E6 but not in C6/36 cells. An overview of CPE formation, with respect to differences observed in the infected cell lines for each strain, is provided in Table S2. While the epidemic strain showed higher viral genome replication rates and reached the plateau after 2 or 3 days post-infection (dpi), M5937-UG-2018 did not reach the plateau after 4 dpi in most cell lines (Fig. 3A). M5937-UG-2018 and SA H177 both replicated to similar levels only in C6/36 cells. In addition, genome copy numbers of M5937-UG-2018 were up to 10-fold lower than for SA H177 in all cell lines except C6/36 (Fig. 3A). Significant differences in the number of viral genome copies between the two strains were found in mosquito (C6/36 and CXT), sheep (Llu-L), human (HEK 293-T), rodent (BHK-21), primate (Vero E6), and chicken (DF-1) cells. We further analyzed the release of infectious particles for both strains in cell culture supernatants of all tested cell lines at 2–4 dpi measured by Tissue Culture Infectious Dose_50_ (TCID_50_)titration. Since viruses did not consistently induce CPE, virus replication in the serial dilutions was assessed by real-time PCR and used as a criterion to classify each well as infected or not infected. Overall, similar differences between M5937-UG-2018 and SA H177 were observed in the release of infectious particles (Fig. 3B). The numbers of M5937-UG-2018 infectious particles in Vero cells were higher than expected based on the genome replication rates. Comparison of the mean viral genome copy numbers of all cell lines in which replication occurred for both strains via two-sample unpaired Student’s *t*-test revealed significant differences between the two strains at 2 dpi (*P* = 0.038) and 3 dpi (*P* = 0.047) (Fig. 4). In summary, M5937-UG-2018 showed differences in the ability to infect cell lines, in the production of viral genome copies, and in the release of infectious particles compared to SA H177.

## DISCUSSION

In the present study, we detected WSLV in a female *Ae. mcintoshi* mosquito collected within the primary rainforest of Semuliki National Park, Uganda. This finding may provide evidence that WSLV is endemic in Uganda and beyond, as the Semuliki rainforest is contiguous with the pristine lowland rainforest of the Congo basin in the Democratic Republic of Congo. WSLV is known to be endemic in Southern Africa, where it is associated with high mortality rates and abortions in newborn and pregnant ruminants, particularly in sheep (3, 8, 32). Although the disease in livestock has not been reported elsewhere, studies conducted in the 1960s–1980s suggest that WSLV also occurs in East and West Africa (19, 20, 33, 34). However, diagnostic techniques at that time mostly relied on serodiagnosis using hemagglutination-inhibition, complement-fixation, and virus neutralization, which makes confidential differential diagnosis nearly impossible due to cross-reactivity among and coinfections with closely related viruses, e.g., yellow fever virus (3, 35). Further evidence of a wider circulation of WSLV in Africa came from more recent studies providing WSLV sequence information from mosquito samples in rural regions in Senegal, Kenya, and Uganda (9, 24, 25). Consequently, these findings indicate that the endemic circulation of WSLV extends beyond Southern Africa, suggesting a wider geographic spread. It has been suggested that larger outbreaks of WSLV do not occur in ruminants in warmer and more humid areas of Southern Africa, as infections are likely to occur throughout the year and induce high immunity levels in the animal population (3, 4, 36–39). This might also explain why no WSLV outbreaks have been reported in Uganda, a region characterized by its largely tropical climate. However, there is currently no information available on antibody prevalence against WSLV in ruminants from Uganda that would support this hypothesis. Systematic seroprevalence studies combined with differential molecular diagnostics in animals presenting with symptoms compatible with a WSLV infection would be necessary to shed light on the actual WSLV geographic distribution and its incidence. As WSLV infection induces symptoms similar to an infection with Rift Valley fever virus or other mosquito-borne infections such as Bunyamwera virus, diagnosis based on symptom presentation is not sufficient for the identification of the causative agent.

Another reason for the absence of outbreaks of abortions, teratology, and neonatal deaths in ruminants in Uganda may also be that M5937-UG-2018 is less pathogenic. To get a first phenotypic assessment of M5937-UG-2018, its infectivity and production of viral genome copies and infectious particles in a range of different cell lines were compared to the prototype WSLV strain SA H177 that was isolated from the blood of a field worker in South Africa in 1955 (4). *In vitro* infection experiments using different mosquito, livestock, and human and non-human primate cell lines revealed significant differences between M5937-UG-2018 and SA H177, suggesting that M5937-UG-2018 shows different phenotypic properties. We identified 26 amino acid substitutions between M5937-UG-2018 and SA H177, which are likely to contribute to the differential replication patterns and host tropism. However, since SA H177 has been passaged in cell culture at least six times, it may have acquired adaptive mutations that increased its susceptibility to a broader range of host cells and enhanced production of viral genome copies and infectious particles. Further epidemiological field studies and defined laboratory infection experiments including reverse genetics would be needed for a more thorough assessment.

We further investigated the possibility that M5937-UG-2018 is not endemic to Uganda but rather was introduced from Southern Africa by reconstructing the phylogeographic spread of WSLV. A recent introduction of WSLV to East Africa would also explain the absence of associated symptoms of disease. While the phylogeographic model estimated an introduction of WSLV to East Africa in the early 2000s, alterations of the geographic origin of our isolate to different locations in West, Central, and South Africa did not reduce the likelihood of the analyses supporting the hypothesis of a wider distribution of WSLV and of an endemic circulation of WSLV in Semuliki National Park. Bayes factor analysis showed that the data do not support the hypothesis that M5937-UG-2018 was introduced into the sampling area. However, the limited availability of WSLV genome sequences has hindered a robust understanding of its evolutionary dynamics and spread. The Bayesian model of phylogeographic diffusion implemented in this study favors exhaustive genomic data for an accurate and robust inference of geographic spread and evolutionary history (40). For a more comprehensive phylogeographic analysis of WSLV, additional sequence data from regions outside Southern Africa would be necessary.

In conclusion, our findings highlight the existence of WSLV in Uganda, likely maintained via an enzootic (sylvatic) amplification cycle. To get a better understanding of the extent and impact of WSLV across the African continent, it would be crucial to conduct further studies focusing on WSLV infection, symptoms, and seroprevalence rates in human as well as domestic and wild animals.

## MATERIALS AND METHODS

### Mosquito collection

Mosquitoes were sampled at three distinct sites within Semuliki National Park and three sites outside the park in western Uganda in 2018 and 2019. The sampling sites were situated at a minimum distance of 1 km from each other and at least 500 m away from the National Park boundary. This boundary marked the transition from the tropical lowland rainforest that is continuous with the rainforest of the Congo basin in the Democratic Republic of Congo to a disturbed habitat characterized by small-scale agriculture. We used Biogents Sentinal 2 (9×), CDC Light traps (9×), and CDC gravid traps (4×), which were baited with a total of six different attractants and placed along two parallel transects with a minimum distance of 20 m. Sampling was conducted at each site for 5 consecutive days and nights in both 2018 and 2019. Individual mosquito specimens were identified morphologically (41, 42) and, where necessary, confirmed via cytochrome oxidase I PCR using primers LCO1490 and HCO2198 (43) followed by Sanger sequencing.

### Virus screening

After mosquito homogenization using ceramic beads and a Qiagen TissueLyser, pools were created from clarified homogenates of 10 conspecific mosquitoes, respectively. RNA was extracted using a Roche MagNA Pure 96 instrument according to the manufacturers’ instructions. Viral RNA was transcribed into cDNA using Invitrogen SuperScript IV Reverse Transcriptase and superpools created from cDNA of eight pools. Superpools were screened on a Roche LightCycler 480 with a WLSV-specific qPCR using a newly designed primer set:

forward: 5′-GTGGAAGGAACAGGCTTACAGTA-3′,

probe: 5′-TGGCTACATACTGAAGGAACTGGGTGGCA-3′,

reverse: 5′-GTGTCATCTGCATACATGTTTCCTC-3′.

### Whole-genome sequencing

The library for high-throughput sequencing was prepared using the Roche KAPA RNA HyperPrep Kit. Quality control was carried out on an Agilent Tapestation 4200, and sequencing was performed on an Illumina MiSeq using the 600-cycle Reagent Kit (version 3). Reads were quality-trimmed and mapped to a WSLV reference sequence (NC_012735) using Geneious Prime 2023.1.2 under default settings.

### Phylogenetics

All available WSLV sequences, 7 complete WSLV genome sequences, and 18 partial NS5 sequences (length = 952 nt), were downloaded from the NCBI nucleotide database (44). A list containing accession numbers, strain information, and the date and location of sampling for all sequences used can be found in Table S3 in the supplemental appendix. A sequence alignment of the WSLV strain and all other available WSLV sequences identified here was generated using Clustal Omega (45), and the best-fit nucleotide substitution model was selected based upon the Bayesian information criterion of the model test performed in MEGA (version 11) (46). A phylogenetic tree was reconstructed running MrBayes (47) for 10,000,000 generations on four MCMC chains with subsamples taken every 1,000 generations. The resulting tree topology was verified by calculation of a maximum likelihood tree inference using IQ-TREE (version 2.1.2) (29) under default automatic model selection parameters and with complete, non-parametric bootstrapping of 1,000 replicates.

### Selection pressure

An alignment of the coding sequences of the eight available complete WSLV genomes was created using MAFFT (version 7.490) (E-INS-I, 1PAM / *k* = 2) (48). To identify which parts of the genome underlie episodic diversifying selection pressure, the HyPhy method MEME (*P* < 0.05) (30) and the aBSREL model (*P* < 0.05) (31) were executed under default settings.

### Phylogeography

For the phylogeographic inference of WSLV diffusion through time and space, the above-described sequence data set comprising all available WSLV sequences (8 complete and 18 partial NS5 WSLV sequences) was used (Table S3). The geographic coordinates for each sampling location were obtained from the original publications or database entries associated with the sequence. Phylogeographic analysis was performed using the Bayesian Evolutionary Analysis Sampling Trees (BEAST) software (version 1.10.4) (49). The best nucleotide substitution model, TN93 + G + I (50), was identified using MEGA (version 11). Since there was no evidence of rate variation between lineages of the data set, we used a strict molecular clock. Constant coalescent (51) was chosen as tree prior based on our assumption of a constant effective population size over time. The Markov chain Monte Carlo (MCMC) analyses were run for 100 million generations, with sampling every 10,000 generations. Tracer (version 1.7.3) (52) was used to ensure that all relevant parameters achieved adequate statistical mixing and sufficient estimated sample sizes. The maximum clade credibility (MCC) tree was selected from the posterior distribution using TreeAnnotator (version 1.10.4) (49) after discarding the first 10% of the samples as burn-in. The MCC tree was visualized and annotated using the spatiotemporal reconstruction of evolutionary dynamics tool (spreaD3 version 0.9.7) (53).

Arbitrary coordinates from various locations across Africa, as well as Berlin, Germany, were used to alter the geographic coordinates of the novel strain in 11 different Bayesian phylogeographic models implemented in BEAST. We then computed the marginal likelihood estimates of each model using the stepping-stone sampling method and compared them to the estimates of the correct placement using Bayes factors.

### Virus isolation and growth analyses

Virus isolation of M5937-UG-2018 was conducted using clarified supernatant of the mosquito homogenate for infection of C6/36 cells (*Aedes albopictus*, RRID:CVCL_Z230) (54) as described previously (55). Cells were either infected with a filtrated solution (0.45 µm) or with the addition of an antibiotic agent containing 1% penicillin/streptomycin and 1% nanomycopulitin to the cell culture media. Virus isolations from both attempts were successful. A stock was generated from the second passage of the cell culture supernatant containing antibiotics, and the virus titer was determined via TCID_50_ titration in C6/36 and Vero E6 cells (56). Virus positive wells were verified via real-time PCR with the aforementioned primers. As reference, a viral stock of WSLV strain SA H177 (UVE/WESSV/UNK/ZA/SAH177 99871–2) obtained from the European Virus Archive was prepared in Vero E6 cells (RRID:CVCL_0059). The genomes of M5937-UG-2018 and SA H177 from the viral stocks were sequenced and compared to reference sequences. Virus titers were determined via TCID_50_ titration in C6/36 and Vero E6 cells, respectively, and virus replication for each well was assessed by real-time PCR. All wells showing an increase in genome copies were considered as virus positive. The titers of M5937-UG-2018 were consistently two logs lower in both cell lines compared to that of SA H177. The titers based in C6/36 titration for each virus isolate were used to estimate the multiplicity of infection (MOI) for the growth kinetics. Virus growth kinetics were performed by infection of C6/36 (*Aedes albopictus*), CXT (*Culex tarsalis*, RRID:CVCL_C4MT), BHK-21 (juvenile hamster kidney, RRID:CVCL_1915), DF-1 (chicken fibroblast, RRID:CVCL_0570), HEK 293-T (human embryonic kidney cells, RRID:CVCL_0063), Llu-L (sheep lung, RRID:CVCL_RX43), Vero E6 (African green monkey, kidney), and ZN-R (goat kidney, RRID:CVCL_0I67) cells with an MOI of 0.1. The following cell lines were obtained from the American Type Culture Collection: Vero E6 (CRL-1586), C6/36 (CRL-1660), HEK 293-T (CRL-3216), BHK-21 (CCL-10), and DF-1 (CRL-3586). Llu-L was kindly provided by Matthias Lenk at the Friedrich-Loeffler-Institute in Greifswald, Germany. CXT was graciously received from Ronald Van Rij at Radboud University Medical Center in Nijmegen, the Netherlands, and the ZN-R cell line was kindly provided by Isabella Eckerle from the University of Geneva, Switzerland. The cell culture supernatant was sampled in 25-µL aliquots every 24 h for 4 consecutive days, and RNA was extracted with a Roche MagNA Pure 96. For cDNA synthesis, Invitrogen SuperScript IV was used with random hexamer primers. Viral RNA copy numbers were quantified via qPCR as described above. Infectious particles in cell culture supernatants at the indicated time points were determined by TCID_50_ titration as described above. Statistical analyses and plotting of the growth curves were performed using R (version 4.3.1).

## Data Availability

Sequence data are available under the GenBank accession number PP333214.

## SUPPLEMENTAL MATERIAL

Supplemental tables and figures spectrum.00914-24-s0001.docx Tables S1 and S2; Fig. S1.

ASM does not own the copyrights to Supplemental Material that may be linked to, or accessed through, an article. The authors have granted ASM a non-exclusive, world-wide license to publish the Supplemental Material files. Please contact the corresponding author directly for reuse.
