# Supplementary material for: Genotypic and phylogeographic insights into a pre-epidemic variant of Wesselsbron virus detected in sylvatic Aedes mcintoshi from Semuliki Forest, Uganda
Source: Microbiol Spectr. 2024 Nov 12;12(12):e00914-24. doi: 10.1128/spectrum.00914-24 (PMC11619370; doi:10.1128/spectrum.00914-24)
Supplement: Supplemental tables and figures — Tables S1 and S2; Fig. S1. [file spectrum.00914-24-s0001.docx]

**Supplementary Material**

**Table S1**: Pairwise nucleotide (blue) and amino acid (red) identities of M5937-UG-2018 (bold) and other WSLV strains.

| **% nt ID** ~ **%** **aa ID** | SA H177 | SA999 | **M5937** | UVRI5236 | AV259 | IP248525 | IP259570 | IP262451 |
| --- | --- | --- | --- | --- | --- | --- | --- | --- |
| SA H177 (EU707555) |  | 98.9 | **99.1** | 99.0 | 98.9 | 98.9 | 98.9 | 98.9 |
| SA999 (MK163943) | 93.2 |  | **99.6** | 99.5 | 99.4 | 99.4 | 99.4 | 99.4 |
| **M5937-UG-2018 (PP333214)** | **93.3** | **97.0** |  | **99.7** | **99.7** | **99.7** | **99.7** | **99.7** |
| UVRI5236 (ON157055) | 93.4 | 97.6 | **97.3** |  | 99.5 | 99.5 | 99.5 | 99.5 |
| AV259 (JN226796) | 93.6 | 97.6 | **98.1** | 97.7 |  | 100 | 100 | 100 |
| IP248525 (KY056256) | 93.3 | 97.1 | **97.5** | 97.2 | 98.2 |  | 100 | 100 |
| IP259570 (KY056258) | 93.4 | 97.0 | **97.4** | 97.2 | 98.2 | 99.8 |  | 100 |
| IP262451 (KY056257) | 93.3 | 97.0 | **97.5** | 97.2 | 98.2 | 99.7 | 99.6 |  |

**Table S2:** Induction of CPE by WSLV strains SA H177 and M5937-UG-2018 on various cell lines. Each cell line was monitored for the presence of CPE, and the dpi at which CPE was first observed is depicted.

| Cell line | SA H177 | M5937-UG-2018 |
| --- | --- | --- |
| C6/36 | - | 3 dpi |
| CXT | - | - |
| Llu-L | 3 dpi | - |
| ZN-R | 4 dpi | - |
| HEK 293-T | - | - |
| Vero E6 | 3 dpi | - |
| BHK-21 | 1 dpi | 1 dpi |
| DF-1 | 3 dpi | - |


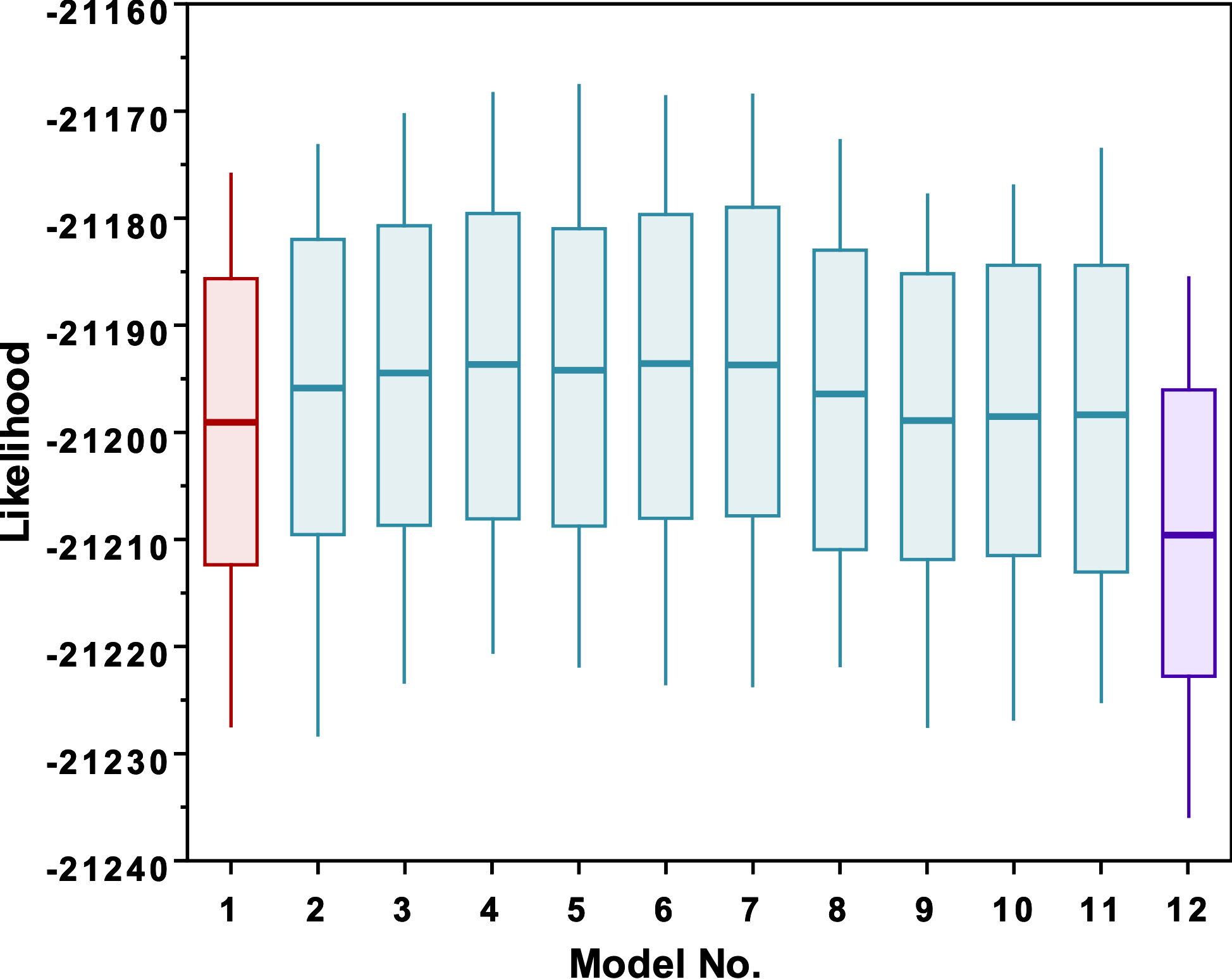


**Figure S1:**

Likelihood values for twelve models traced from Bayesian phylogeographic analysis runs. Geographic coordinates of the novel WSLV strain were altered between the models according to the locations mentioned in Table 4 with the baseline model highlighted in red. Fictional locations of WSLV M5937-UG-2018 in Africa are shown in 2 – 11, and in Berlin, Germany in 12, highlighted in purple.

**Data availability**

**Table S3:** WSLV sequence data used for phylogenetic and phylogeographic analysis. Asterisks behind the accession numbers indicate complete genome sequences. Information about sampling date and location for each strain was derived from the original publications, inquired from the authors or obtained from NCBI meta data.

| **WSLV Strain** | **Accession** | **Country** | **Year** | **latitude** | **longitude** |
| --- | --- | --- | --- | --- | --- |
| VanTonder | JX423772 | South Africa | 1955 | -27.83 | 26.37 |
| TAR100 | JX423788 | South Africa | 1955 | -26.96 | 32.83 |
| SA H177 | DQ859058* | South Africa | 1955 | -27.03 | 32.79 |
| AAR778 | JX423775 | South Africa | 1957 | -25.77 | 29.46 |
| AN2351 | JX423789 | South Africa | 1957 | -25.77 | 29.46 |
| AR748 | JX423773 | South Africa | 1957 | -25.77 | 29.46 |
| AR814 | JX423776 | South Africa | 1957 | -26.93 | 32.28 |
| AR750 | JX423774 | South Africa | 1957 | -25.77 | 29.46 |
| AR2114 | JX423778 | South Africa | 1959 | -26.93 | 32.28 |
| AR2209 | JX423777 | South Africa | 1959 | -26.93 | 32.28 |
| AR3132 | JX423779 | South Africa | 1960 | -26.93 | 32.28 |
| H1028 | JX423786 | South Africa | 1966 | -26.93 | 32.28 |
| AR9512 | JX423791 | South Africa | 1967 | -26.93 | 32.28 |
| AN16210 | JX423783 | South Africa | 1968 | -32.26 | 24.55 |
| AR11173 | JX423780 | Zimbabwe | 1969 | -17.63 | 30.95 |
| AR11189 | JX423781 | Zimbabwe | 1971 | -17.63 | 30.95 |
| AR11190 | JX423782 | Zimbabwe | 1972 | -17.63 | 30.95 |
| AV259 | JN226796* | South Africa | 1996 | -28.29 | 26.15 |
| SA999 | MK163943* | South Africa | 2010 | -27.18 | 25.33 |
| AMH007050 | KM088034 | Kenya | 2010 | -4.27 | 39.43 |
| SPU195 | JX423790 | South Africa | 2011 | -30.72 | 25.10 |
| IP248525 | KY056256* | Senegal | 2013 | 12.63 | -12.42 |
| IP259570 | KY056258* | Senegal | 2013 | 12.56 | -12.18 |
| IP262451 | KY056257* | Senegal | 2013 | 12.77 | -12.23 |
| UVRI5236 | ON157055* | Uganda | 2016 | -1.23 | 30.00 |
| M5937-UG-2018 | PP333214* | Uganda | 2018 | 0.82 | 30.15 |
